# Prognostic Significance of Vitamin D Receptor Polymorphisms in Head and Neck Squamous Cell Carcinoma

**DOI:** 10.1371/journal.pone.0029634

**Published:** 2011-12-29

**Authors:** Takanori Hama, Chihiro Norizoe, Hiroaki Suga, Takeshi Mimura, Takakuni Kato, Hiroshi Moriyama, Mitsuyoshi Urashima

**Affiliations:** 1 Division of Molecular Epidemiology, Jikei University School of Medicine, Tokyo, Japan; 2 Department of Oto-Rhino-Laryngology, Jikei University School of Medicine, Tokyo, Japan; 3 Meiji Pharmaceutical University, Tokyo, Japan; 4 Department of Pediatrics, Jikei University School of Medicine, Tokyo, Japan; National Cancer Center, Japan

## Abstract

**Background:**

In patients with advanced non-small-cell lung cancer, vitamin D receptor (VDR) polymorphisms and haplotypes are reported to be associated with survival. We hypothesized that a similar association would be observed in patients with head and neck squamous-cell carcinoma (HNSCC).

**Methods:**

In a post-hoc analysis of our previous prospective cohort study, VDR polymorphisms including Cdx2 G/A (rs11568820), Fok*I* C/T (rs10735810), Bsm*I* A/G (rs1544410), Apa*I* G/T (rs7976091), and Taq*I* T/C (rs731236) were genotyped by sequencing in 204 consecutive patients with HNSCC who underwent tumor resection. Progression-free survival was compared between VDR polymorphisms using Kaplan-Meier survival curves with log-rank tests and Cox proportional hazard models adjusting for age, gender, smoking status, primary tumor sites, postoperative stages, existence of residual tumor, and postoperative treatment with chemotherapy or radiotherapy.

**Results:**

During a median follow-up of 1,047 days, tumor progression and death occurred in 76 (37.3%) and 27 (13.2%) patients, respectively. The Fok*I* T/T genotype was associated with poor progression-free survival: median survival for T/T was 265 days compared with 1,127 days for C/C or C/T (log-rank test: *P* = 0.0004; adjusted hazard ratio, 3.03; 95% confidence interval, 1.62 to 5.67; *P* = 0.001). In contrast, the other polymorphisms (Cdx2, Bsm*I*, Apa*I*, Taq*I*) showed no significant association with progression-free survival. The A-T-G (Cdx2-Fok*I*-Apa*I*) haplotype demonstrated a significant association with a higher progression rate (*P* = 0.02).

**Conclusion:**

These results suggest that VDR polymorphisms and haplotypes may be associated with prognosis in patients with HNSCC, although the sample size is not large enough to draw definitive conclusions.

## Introduction

Many ecological studies demonstrate associations between UVB rays and a lower risk of developing various cancers except skin cancer, implying that UVB rays induce vitamin D synthesis, which may suppress growth and induce differentiation/apoptosis of cancer cells [1]. A plausible explanation for why higher circulating levels of vitamin D are associated with a decreased risk of deadly cancers is as follows [2]. Epithelial cells convert the primary circulating form of vitamin D, 25-hydroxyvitamin D (25(OH)D), to its active form, 1,25-dihydoroxyvitamin D, inside the cells, which bind vitamin D receptors (VDR) in their cytoplasm to regulate a variety of genes. These genes prevent malignant transformation by keeping cellular proliferation and differentiation within normal ranges. In turn, if a cell becomes malignant, 1,25-dihydroxyvitamin D can induce apoptosis and prevent angiogenesis, thereby reducing the potential for the malignant cell to survive. Recently, vitamin D has been proven to regulate molecules related to the cell cycle and apoptosis *in vitro* [3], [4], *in vivo* [5], [6], and in a pilot randomized double-blind, placebo-controlled clinical trial [7].

Observational studies have suggested inverse associations between serum levels of 25(OH)D and incidence rates of colon, breast, ovarian, renal, pancreatic, aggressive prostate, and other cancers [8], [9]. Moreover, VDR Fok*I* and Bsm*I* single nucleotide polymorphisms (SNPs) might modulate the risk of breast, skin, and prostate cancer as well as other cancer sites [10], [11]. To prove the efficacy of vitamin D in primary cancer prevention, double-blind randomized placebo-controlled trials are needed. Higher doses (1,100IU) of vitamin D plus calcium were shown to significantly reduce cancer incidence [12], although lower doses (400 IU) of vitamin D did not decrease the incidence of colorectal cancer or breast cancer [13], [14].

Although there are many studies investigating associations between serum 25(OH)D levels/VDR polymorphisms and risks of developing cancer, only a few studies have examined the impact of serum 25(OH)D levels on the prognosis of patients with cancer [15]–[18]. Recently, Heist et al. demonstrated that the T allele of VDR Fok*I* polymorphism and the G-T-C (Cdx2-Fok*I*-Bsm*I*) haplotype are associated with significantly worse survival in patients with advanced non-small-cell lung cancer; this was a report to show a relationship between VDR polymorphisms and prognosis of patients with cancer [19].

We hypothesized that associations between VDR polymorphisms and prognosis may be observed not only in specific cancers such as breast and lung cancer but also in other kinds of cancers. Although a variety of VDR polymorphisms were reported to be associated with different disease phenotypes in previous studies, the functional effects of the VDR polymorphisms Cdx2 and Fok*I* have been confirmed [20]. In this study, we assessed associations between five polymorphisms (Cdx2, Fok*I*, Bsm*I*, Apa*I*, and Taq*I*) and progression-free survival in patients with head and neck squamous cell carcinoma (HNSCC).

## Methods

### Patients

This was a post-hoc analysis of VDR polymorphisms in our prospective cohort study up to February 2008 [21] plus an extension up to August 2011. The study was approved by the Ethics Committee for Biomedical Research of the Jikei Institutional Review Board, Jikei University School of Medicine, Tokyo, Japan. All patients provided written informed consent. Between September 2006 and August 2011, tumors and peripheral blood samples were obtained from HNSCC patients who underwent surgery at the Department of Head and Neck Surgery, Jikei University Hospital. A total of 204 consecutive patients with HNSCC who underwent tumor resection were included in this study. Clinical information was obtained from clinical and surgical charts until September 2011. Based on postoperative staging, tumor node metastasis (TNM) classification and cancer stages were determined according to the 6th Union for International Cancer Control TNM classification and stage groupings. Existence of residual tumor and postoperative chemoradiotherapy was also checked to use in multivariate analysis. Patients were periodically (every 0.5 to 2 months) examined on an outpatient basis to make sure they had not progressed. Smoking index was defined as the number of cigarettes smoked daily multiplied by the number of years of smoking. Periodic examinations consisted of standard tests, including endoscopy and computed tomography of the chest and neck.

### Samples

In each case, tumor samples from the primary site and surrounding normal tissue, but not metastatic sites, were stored at −80°C after excision. Cancer tissue was divided into 2 specimens: 1 for pathological confirmation, where the sample was composed of more than 70% cancer cells, and the other for DNA extraction. Peripheral blood samples were collected during the preoperative period from the same patients to confirm whether alteration of the sequence was due to genomic polymorphisms or somatic mutations.

### Polymorphisms of the VDR gene

DNA was extracted from the fresh frozen tumor and peripheral blood samples using QIAcube (Qiagen, Tokyo, Japan) following the manufacturer's protocol. DNA fragments including Fok*I* and Cdx2 were amplified by polymerase chain reaction (PCR) using the following primers (forward/reverse): for Fok*l* (rs10735810), ctccgaaggcactgtgctcaggcct/atggaaacaccttgcttcttctccctc; sequence, ggcctgggccctggggagat; parameters: denaturation at 98°C for 1 min, followed by 30 cycles at 98°C for 10 s, annealing at 68°C for 4 min, and the stopping reaction at 16°C. For Cdx2 (rs10735810), gggaaggagggagggaggaaggaagg/agctgtagcaatgaaagcaaacc; sequence, tagaaaacattgtagaacatc; parameters: denaturation at 95°C for 3 min, followed by 30 cycles at 95°C for 90 s, annealing at 59°C for 90 s, then at 72°C for 2 min, and 16°C. For Bsm*I* (rs1544410), gctgagggccagctgggcaacctgaa/aaccagcgggaagaggtcaaggg; sequence, gggcaacctgaagggagacgtagc; parameters: denaturation at 94°C for 3 min, followed by 35 cycles at 94°C for 20 s, annealing at 62°C for 40 s, extension at 72°C for 1 min, then final extension at 72°C for 6 min, and 16°C. For Apa*I* (rs7976091) and Taq*I* (rs731236), agagcatggacagggagcaaggccaggcag/gcgcaggtcggctagcttctggatcatc: sequence, agagcatggacagggagcaaggccaggcag; parameters: denaturation at 94°C for 10 min, followed by 35 cycles at 93°C for 45 s, annealing at 66°C for 30 s, extension at 72°C 45 s, then final extension at 72°C for 10 min, and 16°C.

The PCR products were incubated with Rapid alkaline phosphatase (Roche Diagnostics, Mannheim, Germany) and Exonuclease I (New England BioLabs, Ipswich, MA) at 37°C for 30 min, then 80°C for 15 min. Using Big Dye Terminator v3.1 Cycle Sequencing kit (Applied Biosystems, Tokyo, Japan), the aliquots were incubated under the following conditions: 96°C for 5 min; 25 cycles of 96°C for 10 s, 50°C for 5 s, 60°C for 2 min. Treated PCR products were sequenced with the ABI PRISM 3700 Genetic Analyzer (Applied Biosystems, Foster City, CA). Sequencing was confirmed with independent duplicate analyses.

### Statistical analysis

Mann-Whitney test and chi-square test were used to evaluate differences in patients' characteristics stratified by VDR genotypes. Progression-free survival was defined as the length of living time after surgery during which no apparent progression of cancer was found; curves were drawn using the Kaplan-Meier method and compared by VDR polymorphisms using log-rank tests. Cox proportional hazard models were fitted for multivariate analysis adjusting for age, gender, smoking status, primary tumor sites, postoperative stage (stage I to stage IV), existence of residual tumor, and postoperative treatment with chemotherapy or radiotherapy. Adjusted hazard ratios (HR) and 95% confidence intervals (CI) were computed. All statistical analyses were performed using STATA 12.0 (STATA Corp., College Station, TX). P<0.05 was considered statistically significant.

Hardy-Weinberg equilibrium was assessed by the chi-square test. Linkage disequilibrium relationships of VDR SNPs that were not significant as assessed with Hardy-Weinberg equilibrium were visualized based on calculating D′. Genotype and allele frequencies were compared between progressed and non-progressed patients by means of chi-square analysis. Global differences in haplotype frequencies between progressed and non-progressed patients were assessed using 10,000 permutations for each comparison to correct for inaccurate p-values due to multiple comparisons. All analyses were done using Haploview [22].

## Results

### Patients' characteristics and VDR polymorphisms

Patients' age ranged from 32 to 89 years, and there were more men than women. More than half of patients (55%) were smokers; 64% of smokers were men. Most patients had oropharyngeal cancer, and 51% had stage IV disease. In 10 patients, tumor tissue was not resected completely. Thirty patients underwent chemotherapy or radiotherapy after surgery.

**Figure 1:**
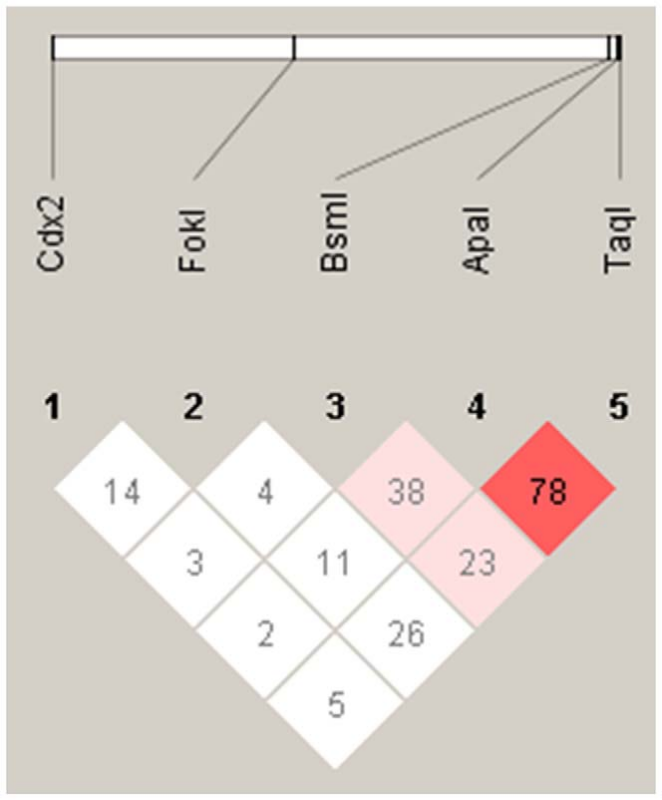
Linkage disequilibrium plot across vitamin D receptor gene in 172 patients with HNSCC. Numbers within the diamonds are D′ values for the respective SNP pairs.

We confirmed that polymorphisms were common between the tumor and peripheral blood samples obtained from the same patients. Genotype and allele frequencies of the VDR polymorphisms are shown in Table 1. Hardy-Weinberg equilibrium test was significant for Bsm*I* (*P* = 2.7×10^−5^) and Taq*I* (*P* = 0.04). Linkage disequilibrium analysis between VDR polymorphisms was shown (Fig. 1). D′ between Apa*I* and Taq*I* was 78. Patients' characteristics stratified by Fok*I* genotype are shown in Table 2. No significant differences were observed between patients' characteristics and Fok*I* or other polymorphisms (data not shown).

**Table 1:** Genotype and allele analyses of vitamin D receptor gene polymorphisms.

SNPs	GenotypeAllele	N	HW p-value*1
Cdx2			0.06
rs11568820	AA	28	
(Promoter)	GA	67	
	GG	77	
	A	123	
	G	221	
Fok*I*			0.08
rs10735810	CC	73	
(Exon2)	CT	112	
	TT	19	
	C	258	
	T	150	
Bsm*I*			2.7×10^−5^
rs1544410	AA	12	
(Intoron8)	AG	27	
	GG	133	
	A	51	
	G	293	
Apa*I*			0.31
rs7975232	GG	96	
(Intoron8)	GT	61	
	TT	15	
	G	253	
	T	91	
Taq*I*			0.04
rs731236	TT	129	
(Exon8)	CT	38	
	CC	5	
	T	296	
	C	48	

*1 : Hardy-Weinberg equilibrium was assessed by the chi-square test.

**Table 2:** Patients' characteristics stratified by FokI genotype.

Variable	Total (n = 204)	Fok I C/C C/T (n = 185)	Fok I TT (n = 19)	*P* value
Age (years)	63.7±11.2	63.4±11.5	65.9±8.3	0.45*1
Male/female	157/47	142/43	15/4	0.83*2
Smoking status (pack-year)	20.9±25.7	19.7±24.4	32.4±34.6	0.12*1
Primary site				0.58*2
Oropharyngeal	54	47	7	
Hypopharyngeal	49	45	4	
Laryngeal	36	34	2	
Oral cavity	46	43	3	
Nasal cavity	19	16	3	
T stage: T1/T2/T3/T4	22/89/43/50	21/78/39/47	1/11/4/3	0.53*2
N stage: N0/N1/N2	97/33/74	90/29/66	7/4/8	0.60*2
Stage: I/II/III/IV	14/40/47/103	14/37/42/92	0/3/5/11	0.59*2
Existence of residual tumor	10 (4.8%)	9 (4.9%)	1 (5.3%)	0.94*2
Chemo-radio therapy	30 (14.7%)	28 (15.1%)	2 (10.5%)	0.59*2

*1 : P-value was calculated by Mann Whitney test.

*2 : P-value was calculated by chi-square test.

### VDR polymorphisms and progression-free survival

**Figure 2:**
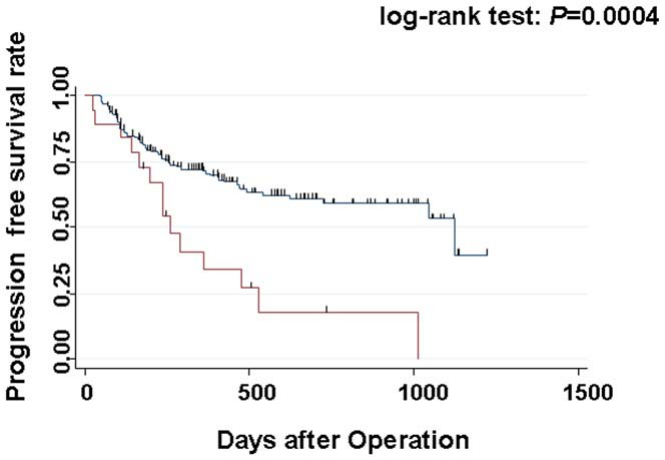
Kaplan-Meier curves of progression-free survival by Fok*I* polymorphism in 204 patients with HNSCC. Difference in time until progression was compared between Fok*I* C/C plus C/T and T/T.

All 204 patients were followed for a median of 1,047 days (range, 23 to 1224 days), during which 76 (37.3%) progressed and 27 (13.2%) died of HNSCC. Kaplan-Meier survival curves showed that the FokI T/T genotype was associated with poor prognosis: median progression-free survival for T/T was 265 days compared with 1,127 days for C/C or C/T (log-rank test: *P* = 0.0004) (Fig. 2). In contrast, the other polymorphisms (Cdx2, Bsm*I*, Apa*I*, and Taq*I*) showed no significant associations with progression-free survival (data not shown).

### Cox proportional hazard models with multivariate adjustment

Cox proportional hazard models were computed to determine the significance of Fok*I* with adjustment for age, gender, smoking status, primary tumor site, postoperative stage, existence of residual tumor, and postoperative treatment with chemotherapy or radiotherapy (Table 3). Without multivariate analysis, the Fok*I* T/T genotype and stages showed a significant crude HR. With multivariate analysis, patients with the Fok*I* T/T genotype showed poor prognostic markers: adjusted HR, 3.03; 95% CI, 1.62 to 5.67; *P* = 0.001. In contrast, the other polymorphisms (Cdx2, Bsm*I*, Apa*I*, Taq*I*) showed no significant association with progression-free survival (data not shown).

**Table 3:** Cox proportional hazard models.

Variable	Crude HR	95% CI	*P* value	Adjusted HR*	95% CI	*P* value
FokI T/T	2.73	1.52–4.89	0.001	3.03	1.62–5.67	0.001
FokI C/C+C/T	Reference			Reference		
Age	0.99	0.97–1.01	0.48	0.98	0.96–1.01	0.14
Male	1.22	0.69–2.14	0.50	1.38	0.75–2.56	0.30
Smoking index	1.00	0.99–1.01	0.58	1.00	0.99–1.01	0.48
Primary site						
Oropharyngeal	Reference			Reference		
Hypopharyngeal	0.98	0.51–1.89	0.96	1.00	0.51–1.97	0.99
Laryngeal	0.98	0.48–2.02	0.96	1.14	0.54–2.42	0.77
Oral cavity	0.98	0.52–1.88	0.96	1.14	0.59–2.24	0.69
Nasal cavity	1.66	0.75–3.68	0.21	1.60	0.71–3.62	0.26
Stages I∼IV	1.54	1.18–2.01	0.002	1.60	1.20–2.15	0.002
Existence of residual tumor	1.18	0.43–3.22	0.75	1.36	0.47–3.91	0.57
Chemoradiotherapy	1.18	0.64–2.19	0.60	1.40	0.73–2.70	0.32

*Adjusted for age, gender, smoking status, primary tumor sites, postoperative stages, existence of residual tumor, and postoperative treatment with chemotherapy or radiotherapy.

HR, hazard ratio; CI, confidence interval.

### VDR haplotype (Cdx2-Fok*I*- Apa*I*) and permutation analyses

Because Hardy-Weinberg equilibrium test was significant for Bsm*I* and Taq*I*, only Cdx2, Fok*I*, and Apa*I* were used in haplotype analyses. Associations between VDR haplotypes and disease progression were analyzed with permutation analysis (Table 4). The A-T-G (Cdx2-Fok*I*-Apa*I*) haplotype demonstrated a significant association with a higher progression rate (*P* = 0.02).

**Table 4:** VDR haplotype (Cdx2, Fok*I*, and Apa*I*) frequencies and permutation analysis*2.

HaplotypeCdx2 -Fok*I* -Apa*I*	Frequency (N)	Chi Square	Permutation*1P-value
ATG	0.171 (29)	4.375	0.0200
ACG	0.299 (51)	2.192	0.4400
GTG	0.075 (13)	1.578	0.6500
GCG	0.191 (33)	0.684	0.9600
ACT	0.093 (16)	0.102	1.0000
ATT	0.080 (14)	0.0070	1.0000
GCT	0.056 (10)	0.267	1.0000
GTT	0.035 (6)	0.075	1.0000

*1 : Number of permutation was 10,000 times.

*2 : Analyses were performed using samples from 172 patients.

## Discussion

In this study, we found that the VDR Fok*I* T/T genotype was associated with a poor progression-free survival rate in patients with HNSCC, even after adjusting for age, gender, smoking status, primary tumor site, cancer stage, residual tumor, and postoperative treatment (chemotherapy or radiotherapy). Arai et al. demonstrated that compared with the Fok*I* T/T genotype, Fok*I* C/C had 1.7-fold greater function of vitamin D-dependent transcriptional activation of a reporter construct under the control of a vitamin D response element in transfected HeLa cells [23]. By switching from the ATG (Fok*I* T) to the ACG (Fok*I* C) polymorphism, the first potential start site moved to the 3′ direction, resulting in proteins that were 3 amino acids shorter and more functional [24], [25]. Thus, patients with Fok*I* T/T may have less response to vitamin D, resulting in a higher progression rate.

The haplotype A-T-G (Cdx2-Fok*I*-Apa*I*) was associated with poor prognosis. This result is similar to previous studies showing that lower serum 25(OH)D levels and the Fok*l* T/T genotype as well as a VDR haplotype (Cdx2-Fok*I*-Bsm*I*) were associated with poor prognoses in colorectal cancer and lung cancer, respectively [15], [19].

There are many reports suggesting that vitamin D can reduce tumor growth of HNSCC *in vitro* and *in vivo*. Activated vitamin D3 analogue EB1089 at nanomolar concentrations completely inhibited growth of HNSCC cells [26]. Using KB cells from an oral floor with squamous cell carcinoma, vitamin D3 was shown to suppress cell proliferation, induce apoptosis and cell cycle arrest, upregulate sensitivity of chemotherapeutic drugs, and downregulate several angiogenesis factors and an apoptotic factor, survivin [27]. Similarly, vitamin D3 analogue inhibited the proliferation of human laryngeal squamous carcinoma cells through the cyclin-dependent kinase inhibitor p57 or p21 [28], [29]. Moreover, systemic vitamin D3 therapy delayed carcinogenesis in the hamster buccal pouch model [30]. Treatment of HNSCC patients with activated vitamin D reduced levels of immune inhibitory CD34 (+) cells while increasing maturation of dendritic cells [31], where a reduced progression rate can be expected [32]. Fok*I* and Taq*I* polymorphisms in VDR were reported to affect the development of HNSCC [33]. These findings are not inconsistent with our results.

There are several limitations to our study. Overall, the sample size is not large enough to detect significant differences of minor VDR polymorphisms or synergistic effects of the gene-environment and stratification by tumor stage or primary site. Although Fok*I* TT was a significant factor, the number was only 17 and 95%CI was sometimes wide. However, when we set number of patients as 175 FokI CC+CT vs. 19 FokI TT polymorphisms, post-hoc power calculations showed 98% along with a hazard ratio of 2.73. Moreover, we did not use restriction fragment length polymorphism analysis and directly sequenced PCR fragments to avoid misclassification as much as possible and to compensate for the disadvantage of the small sample size. No information was available regarding vitamin D intake (dietary or supplemental), sun exposure, or circulating vitamin D levels of patients. Moreover, we did not have clinical information on alcohol use, human papilloma virus, or Epstein-Barr virus infection. There are other kinds of VDR polymorphisms related to disease risks that have been reported in previous articles, and we need to expand our range of analysis in the future.

In conclusion, there were significant associations between shorter progression-free survival time in patients with HNSCC and Fok*I* T/T genotype as well as A-T-G (Cdx2-Fok*I*-Apa*I*) haplotype, although the sample size is not large enough to detect significant differences of minor VDR polymorphisms or synergistic effects of the gene-environment and stratification by tumor stage or primary site.
